# Effect and Safety of Skincare Regimens Containing a Multi‐Molecular Hyaluronic Acid Complex for Recovery After Ablative Fractional CO_2_ Laser: A Prospective, Randomized, Controlled Trial

**DOI:** 10.1111/jocd.70322

**Published:** 2025-07-01

**Authors:** Zongzhou Wu, Xinlin Liang, Qian Yu, Wei Zhang, Yuling Shi

**Affiliations:** ^1^ Department of Medical Cosmetology, Shanghai Skin Disease Hospital, School of Medicine Tongji University Shanghai China; ^2^ Institute of Psoriasis, School of Medicine Tongji University Shanghai China; ^3^ Department of Dermatology, Shanghai Skin Disease Hospital, School of Medicine Tongji University Shanghai China

**Keywords:** ablative fractional CO_2_ laser, clinical trial, hyaluronic acid, skin barrier, skincare, TEWL

## Abstract

**Background:**

Fractional CO_2_ laser is commonly used for various skin issues but often causes side effects like erythema, hyperpigmentation, and prolonged recovery, negatively impacting patients' quality of life.

**Objectives:**

This study evaluates the effect of hyaluronic acid (HA) dressings with different molecular weights (large: medium: low = 2:2:1) on skin barrier repair after CO_2_ fractional laser treatment, providing clinical evidence for post‐procedural skincare.

**Methods:**

In this prospective, randomized, open‐label, controlled trial, patients aged 19–45 who underwent CO_2_ fractional laser treatment were randomly assigned to either the hyaluronic acid (HA) group (Group A) or the control group (Group B). The primary endpoint was transepidermal water loss (TEWL) at day 14. Secondary endpoints included erythema index (EI), melanin index (MI), skin hydration, semi‐quantitative acute inflammatory response scores (erythema, burning sensation, edema), erythema duration, edema duration, pain duration, scab shedding time, and overall patient satisfaction on day 14.

**Results:**

A total of 60 patients were included. On day 14, the TEWL in Group A was significantly lower than in Group B (16.69 vs. 19.79, *p* = 0.009), and the percentage change in TEWL during the period of the most significant reduction was also greater in Group A than in Group B (−75.10% vs. −70.89%, *p* = 0.042). The EI in Group A were significantly lower than those in Group B on days 3, 7, and 14 (325.41 vs. 370.83, *p* = 0.038; 297.77 vs. 338.32, *p* = 0.041; 287.14 vs. 337.38, *p* = 0.004). The pain duration in Group A was also significantly shorter than in Group B (0.20 vs. 0.53, *p* = 0.014). In addition, Group A patients reported higher satisfaction scores on the post‐treatment questionnaires.

**Conclusions:**

This study highlights hyaluronic acid with varied molecular sizes enhances post‐laser recovery by reducing TEWL and erythema, alleviating pain, and promoting healing.

## 1 Background

### 1.1 Fractional Laser and Adverse Reactions

Fractional lasers, particularly CO_2_ lasers with a wavelength of 10 600 nm, exert photothermal effects by targeting water‐rich structures in the skin, including the epidermis, collagen fibers, and blood vessels. This generates thermal effects that promote epidermal regeneration, collagen synthesis, and remodeling, and ultimately lead to wrinkles, tightening skin, shrinking pores, and improving texture. Clinically, it is primarily used for the treatment of conditions such as atrophic scars, photoaging, and wrinkles [1, 2].

However, CO_2_ fractional laser treatment may compromise the integrity of the skin barrier. Its photochemical effects impair intercellular adhesion, disrupting the stratum corneum's “brick‐wall” structure. Thermal damage leads to keratin denaturation, affecting the skin's barrier. Photomechanical effects create mechanical stress, damaging keratinocytes and causing erythema, edema, dryness, and desquamation [3]. While most of these effects self‐repair, improper management can cause long‐term issues like persistent erythema and post‐inflammatory hyperpigmentation (PIH), which may take months to resolve, causing physical and psychological distress for patients [4].

### 1.2 Postoperative Repair After Fractional Laser Treatment

Proper perioperative management is crucial for laser treatments as it can reduce or prevent the occurrence of adverse reactions and improve treatment outcomes. Postoperative care following fractional laser treatment primarily focuses on promoting healing, maintaining moisture, and sun protection, with a strong emphasis on the restoration of skin barrier function. This helps alleviate postoperative skin reactions, prevent skin infections, accelerate tissue repair, and promote wound healing [5]. Expert recommendations for skincare after non‐surgical and surgical procedures suggest several products to speed up recovery, including mineral water, conjugated linoleic acid, vitamin C/vitamin E/ferulic acid, and so on [6]. Therefore, a range of repair products with anti‐inflammatory and moisturizing effects can be beneficial after CO_2_ fractional laser treatment.

### 1.3 Characteristics of Hyaluronic Acid and Its Role in the Skin

Hyaluronic acid (HA) is a non‐sulfated glycosaminoglycan composed of alternating units of D‐glucuronic acid and N‐acetyl‐D‐glucosamine [7]. It is widely distributed in connective tissues and skin [8]. HA not only contributes to the extracellular matrix along with collagen, elastin, and other components, but also interacts with adhesion proteins like CD44 [9] and intercellular adhesion molecule 1 (ICAM‐1) [10] to regulate cellular functions, including signal transduction, inflammation, wound healing, and angiogenesis. Therefore, HA exhibits various pharmacological effects, including anti‐inflammatory, wound healing, immune modulation, anti‐cancer, anti‐diabetic, anti‐aging, and skin repair functions [11].

In the skin, HA plays a crucial role in moisturizing, immunoregulation, and tissue repair [12]. As a natural moisturizing factor, it can absorb up to 1000 times its weight in water. Studies show that HA improves skin hydration by 134% immediately and maintains a 55% increase after 6 weeks of use in patients with facial photoaging [13]. HA has also demonstrated benefits in wound healing, such as reducing skin reactions after radiotherapy [14] and promoting healing in venous ulcers [15].

However, the biological activity of HA is highly dependent on its molecular weight. High‐molecular‐weight HA (HMW‐HA, with a molecular weight of approximately 2 × 10^6^) forms a protective, moisturizing film on the skin. Low‐molecular‐weight HA (LMW‐HA, with a molecular weight not exceeding 1 × 10^6^) penetrates deeper, offering hydration and regulatory effects. Medium‐molecular‐weight HA (MMW‐HA) lies between HMW‐HA and LMW‐HA in terms of size. Oligomeric HA (HA‐o) is a biological polysaccharide fragment formed by recombinant human HA enzyme cleavage followed by special processing [16, 17]. During the early stages of wound healing, HMW‐HA is degraded into LMW‐HA, which initiates inflammatory responses. In the later stages, newly synthesized HMW‐HA helps suppress inflammation, promote tissue repair, and reduce scar formation [18].

In our randomized controlled trial, we aim to further evaluate the efficacy and safety of multi‐molecular‐weight hyaluronic acid dressings in promoting recovery following CO_2_ fractional laser treatment.

## 2 Materials and Methods

### 2.1 Study Subjects

This prospective, randomized, controlled trial was conducted at the Department of Medical Cosmetic Center of Shanghai Skin Disease Hospital in China. Patients aged 18–60 years with Fitzpatrick skin types II‐IV who met the eligibility criteria were enrolled and received CO_2_ fractional laser treatment. Individuals were excluded from the study if they met any of the following conditions: ① currently undergoing or planning to undergo facial medical treatments or aesthetic procedures; ② a history of allergy to hyaluronic acid or a hypersensitivity constitution; ③ history of keloid formation; ④ active facial infections (e.g., herpes simplex virus infections); ⑤ suspicious malignant lesions on the face; ⑥ organic diseases affecting major organs such as the heart, brain, lungs, liver, or kidneys; ⑦ pregnant or breastfeeding women; ⑧ history of psychiatric or mental disorders; ⑨ other conditions that the investigators deemed unsuitable for participation in the study. If participants experienced unexpected adverse reactions, if investigators determined that they could no longer continue with the trial, or if participants voluntarily withdrew for any reason, they were excluded from the study.

Informed consent was signed and obtained from each participant. This study was conducted in accordance with the research protocol approved by the Ethics Committee of Shanghai Skin Disease Hospital (No. 2023‐1 106 140 (scientific)).

### 2.2 Grouping and Interventions

Based on our previous similar clinical studies, the sample size was determined to be 30 subjects per group [19]. Patients were randomly assigned to the hyaluronic acid group (Group A) and the control group (Group B) using an even‐odd method, with 30 patients in each group. General demographic data, including sex and age were compared between the two groups, and no significant differences were found (*p* > 0.05).

**FIGURE 1:**
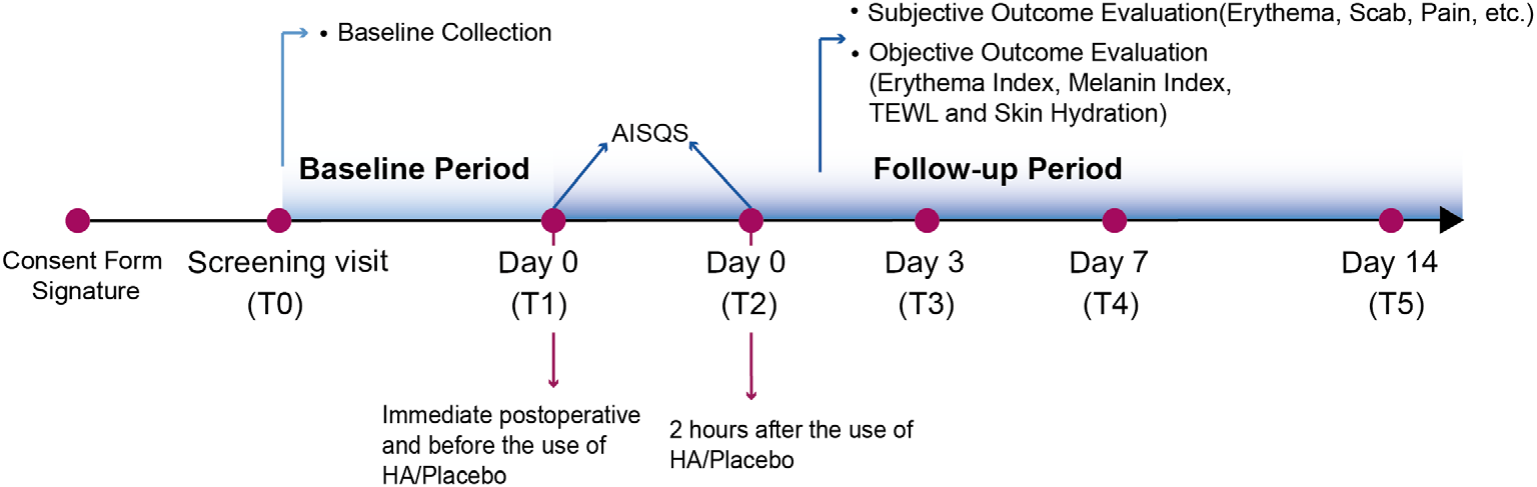
Schematic of study design. The first (T1) and second (T2) follow‐up visits both occurred on day 0. AISQS, Semi‐quantitative score of acute inflammatory response, which were assessed by clinicians; HA, Hyaluronic Acid; TEWL, transepidermal water loss. Subjective evaluations were recorded by the patients.

This study included six visits: screening visit (T0), treatment visit (T1: immediately post‐operation, no product applied), and follow‐up visits (T1–T5: immediately post‐operation, 2 h post‐operation, day 3, day 7, and day 14) (Figure 1). The screening visit (T0), treatment visit (T1: immediately post‐operation), and the first follow‐up visit (T2: 2 h post‐operation) took place on the same day.

At T0, all patients received CO_2_ laser treatment using the CO2RE system. The treatment parameters were set as follows: wavelength: 10.6 μm, energy density: 55 J/cm^2^, spot size: 7.8 × 7.8 mm^2^, frequency: 40 Hz, and coverage: 5%. The device was directly applied to both sides of the face, and after treatment, ice packs were applied to the scar area for 30 min. After 30 min, a hyaluronic acid mask was applied to Group A for 15 min, followed by a placebo cream, while Group B had the placebo cream applied. Data collection for T1 was done 2 h post‐operation for both groups.

For home care, Group A applied the placebo cream for 14 days (twice daily), and used the HA dressing for 7 days, with additional applications on days 8, 10, and 12. Group B applied the placebo cream for 14 days (twice daily). The HA dressing used in this study contained a composite formulation with a molecular weight ratio of large: medium: low molecules = 2:2:1. The placebo was a single cream base (without any special ingredients) formulated by the Shanghai Skin Disease Hospital.

### 2.3 Data Collection and Evaluation

The primary endpoint of this study was the transepidermal water loss (TEWL) measured on day 14. Other evaluation parameters included objective assessments (erythema index (EI), melanin index (MI), and skin hydration on day 14) as well as subjective assessments of treatment (semi‐quantitative score of acute inflammatory response, duration of erythema, duration of edema, duration of pain, scab shedding time, and overall patient satisfaction).

Objective data collection was performed using the multifunctional skin tester MPA20 (Courage Khazaka, Germany) along with the MX18 probe (for EI and MI), the Corneometer CM825 probe (for skin hydration), and the Tewameter TM300 probe (for TEWL).

The semi‐quantitative score of acute inflammatory response (AISQS) was evaluated by clinicians at T1 and T2 based on erythema, burning sensation, and edema at the treatment site. Severity was rated as follows: no erythema, burning, or edema = 0 points; mild erythema, burning, and slight edema = 1 point; moderate erythema and burning with mild edema = 2 points; and marked erythema, burning, and edema = 3 point [20].

The duration of scab shedding, erythema, edema, and pain was recorded daily by the participants. Participant satisfaction was assessed through a self‐reported questionnaire on postoperative day 14.

### 2.4 Data Analysis

For the comparison of baseline data between the two groups for continuous variables, either an independent samples t‐test or the Mann–Whitney *U* test was used based on the data distribution. To account for the influence of baseline levels, comparisons of skin parameters (excluding baseline data) were adjusted using T0 measurements as covariates. Analysis of covariance (ANCOVA) or non‐parametric ANCOVA (Quade Nonparametric ANCOVA) was applied for comparison based on the data distribution, with *p*‐values obtained after Bonferroni adjustment for multiple comparisons. For categorical variables, statistical comparisons were made using the Chi‐square test or Fisher's exact test. All statistical analyses were performed using SPSS (version 29.0), and figures were created using GraphPad Prism (version 10). A significance level of *α* < 0.05 was considered statistically significant in all analyses.

## 3 Results

### 3.1 Baseline Characteristics of Participants

A total of 60 patients were enrolled, all of whom completed the entire treatment protocol and 14‐day follow‐up. The mean (SD) age of group A was 30.37 (6.00) years, and the mean (SD) age of group B was 29.83 (6.01) years. There were no significant differences in gender distribution between the two groups. At baseline, there were no significant differences between group A and group B in terms of EI, MI, skin hydration, or TEWL (Table 1). All participants were included in the following analyses, and the analyses were conducted according to the originally assigned groups.

**TABLE 1:** Baseline characteristics of patients.

	A	B	*p*
*N*	30	30	
Age, years (Mean ± SD)	30.37 ± 6.00	29.83 ± 6.01	0.857
Gender (*n*, %)			
Male	15 (25.00)	15 (25.00)	1.000
Female	15 (25.00)	15 (25.00)	
Skin parameters (Mean ± SD)			
Erythema index	246.06 ± 44.68	270.96 ± 55.87	0.062
Melanin index	94.16 ± 30.95	97.89 ± 35.24	0.664
TEWL	17.25 ± 4.94	17.76 ± 3.87	0.660
Skin hydration	57.87 ± 13.55	53.69 ± 11.32	0.200

### 3.2 Primary Endpoint Comparison—TEWL on Day 14

**FIGURE 2:**
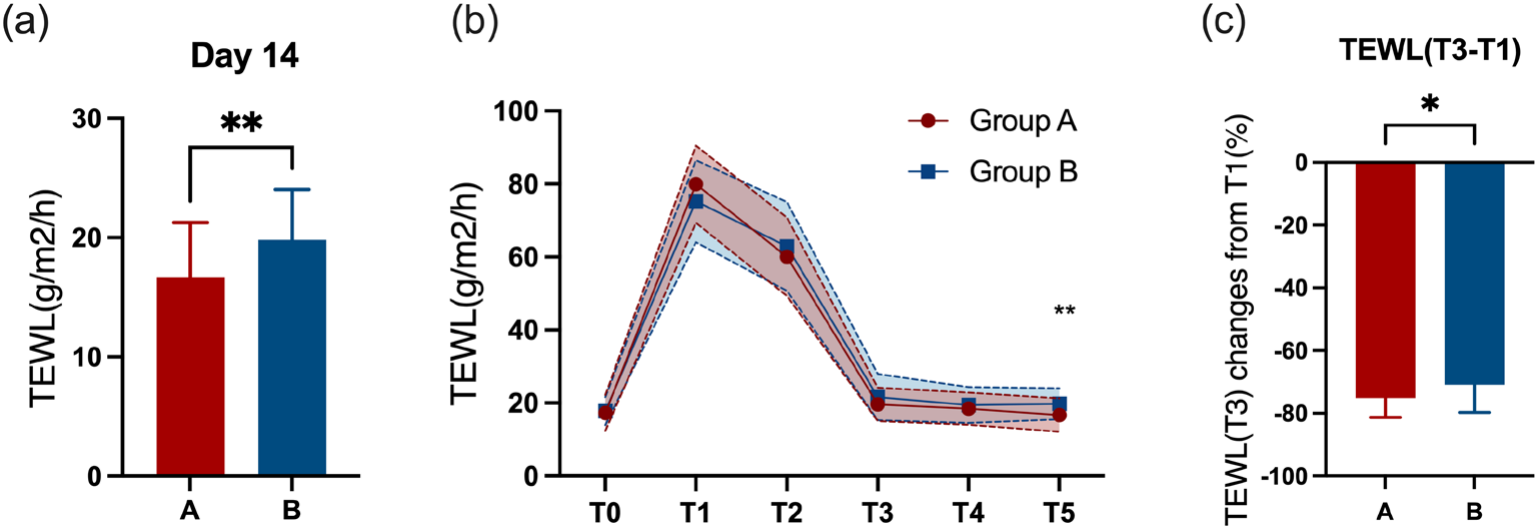
Primary endpoint outcome. (a) TEWL on T5 (day 14). (b) Percentage change in TEWL from T1 (immediate postoperative) to T3 (day 3). (c) TEWL at baseline and during follow‐up. T0: Baseline; T1: Day 0 (immediate postoperative); T2: Day 0 (2 h after the skin care regimen); T3: Day 3; T4: Day 7; T5: Day 14; TEWL, Transepidermal water loss. The red‐ and blue‐shaded areas represent the ranges between the standard deviation added and subtracted to the mean, in hyaluronic acid group and placebo group, respectively. *p < 0.05. **p < 0.01.

On day 14 after CO_2_ fractional laser treatment, the TEWL in group A was significantly lower than that in group B (16.69 vs. 19.79, *p* = 0.009) (Figure 2a). Furthermore, during the period of the most significant decrease in TEWL (T1–T3) (Figure 2b), the percentage reduction in TEWL was greater in group A compared to group B (−75.10% vs. −70.89%, *p* = 0.042) (Figure 2c). These results suggest that HA can accelerate the restoration of hydration during critical periods, thereby contributing to the repair of the skin barrier.

### 3.3 EI, MI, Skin Hydration on Day 14 and All Skin Parameters Changes Relative to Baseline

**FIGURE 3:**
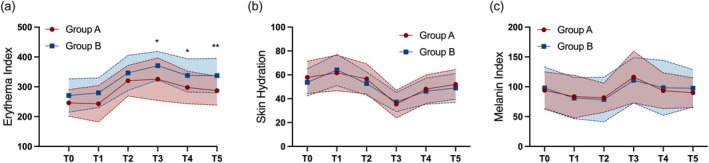
Skin parameters at baseline and during follow‐up. (a) Erythema index; (b) Skin hydration; (c) Melanin index. T0: Baseline; T1: Day 0 (immediate postoperative); T2: Day 0 (2 h after the skin care regimen); T3: Day 3; T4: Day 7; T5: Day 14. The red‐ and blue‐shaded areas represent the ranges between the standard deviation added and subtracted to the mean, in hyaluronic acid group and placebo group, respectively.

In addition to its ability to repair the skin barrier, hyaluronic acid (HA) also significantly improved the erythema index (EI). On postoperative days 3, 7, and 14, the erythema index in the Group A was significantly lower than in the Group B (325.41 vs. 370.83, *p* = 0.038; 297.77 vs. 338.32, *p* = 0.041; 287.14 vs. 337.38, *p* = 0.004) (Figure 3a). However, no significant differences were observed in skin hydration (Figure 3b) or melanin index (MI) (Figure 3c). The *p*‐values are based on data adjusted for covariates, with statistical details presented in Table 2.

**TABLE 2:** Skin parameters comparison results using ANCOVA or Quade nonparametric ANCOVA.

Skin parameters	T1			T2			T3			T4			T5		
	Mean	SE	*p*	Mean	SE	*p*	Mean	SE	*p*	Mean	SE	*p*	Mean	SE	*p*
Erythema index															
A	249.979^a1^	9.036	0.082	—	—	0.333	333.777^a1^	9.406	0.038*	304.765^a1^	8.866	0.041*	295.424^a1^	7.728	0.004**
B	272.909^a1^	9.036		—	—		362.464^a1^	9.406		331.324^a1^	8.866		329.098^a1^	7.728	
Melanin index															
A	85.126^a2^	4.194	0.354	83.036^a2^	4.044	0.340	117.574^a2^	6.045	0.351	—	—	0.712	91.048^a2^	4.226	0.358
B	79.575^a2^	4.194		77.531^a2^	4.044		109.526^a2^	6.045		—	—		96.585^a2^	4.226	
TEWL															
A	79.956^a3^	2.017	0.113	59.959^a3^	2.108	0.325	19.716^a3^	1.008	0.207	—	—	0.572	—	—	0.006**
B	75.356^a3^	2.017		62.924^a3^	2.108		21.537^a3^	1.008		—	—		—	—	
Skin hydration															
A	60.923^a4^	2.480	0.268	55.750^a4^	1.833	0.417	35.151^a4^	1.800	0.339	47.706^a4^	2.096	0.716	51.408^a4^	2.188	0.569
B	64.873^a4^	2.480		53.616^a4^	1.833		37.621^a4^	1.800		46.615^a4^	2.096		49.624^a4^	2.188	

*Note:* *p*‐values were derived from ANCOVA or Quade Nonparametric ANCOVA (depending on the distribution of the effect size). A, B: Estimated marginal adjusted mean based on the covariate of Group A or Group B. a1: Covariates appearing in the model are evaluated at the following values: EI_T0 = 258.5053. a2: Covariates appearing in the model are evaluated at the following values: MI_T0 = 96.0222. a3: Covariates appearing in the model are evaluated at the following values: TEWL_T0 = 17.5080. a4: Covariates appearing in the model are evaluated at the following values: HYD_T0 = 55.7807. “—”: Missing value indicate that this comparison uses Quade Nonparametric ANCOVA and there is no exact estimated marginal value.

Abbreviations: SE, standard error; TEWL, transepidermal water loss.

* *p* < 0.05.

** *p* < 0.01.

**FIGURE 4:**
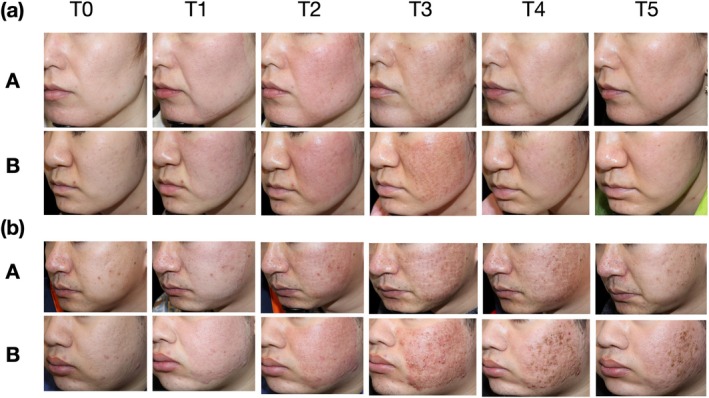
Representative local images showing erythema evolution in selected facial regions at each time point (T0–T5) of the Group A (HA) and Group B (placebo). (a) Female. (b) Male. T0: Baseline; T1: Day 0 (immediate postoperative); T2: Day 0 (2 h after the skin care regimen); T3: Day 3; T4: Day 7; T5: Day 14.

Figure 4 presents representative images of erythema changes at different time points after treatment. Immediately after fractional laser therapy, prominent erythema was observed in the treated areas, reaching its peak at 2 h postoperatively. Subsequently, erythema gradually subsided over time. By day 3, a slight reduction in erythema was noted, although it remained noticeable; at this point (T3), the HA group already demonstrated a visible improvement compared to the control group. By day 7, most erythema had significantly improved; and by day 14, the skin had nearly returned to normal, with only minimal residual redness. However, in Figure 4b, a male patient in the control group still exhibited residual erythema on day 14. These observations are consistent with the EI (erythema index) measurements and further support the efficacy of hyaluronic acid in promoting postoperative erythema resolution.

We then assessed skin repair by comparing the changes from baseline to the endpoint (day 14). Consistent with the results observed on day 14, significant statistical differences were observed in the EI and TEWL in group A compared to group B. The change in TEWL was greater in group A than in group B (−0.56 vs. 2.02, *p* = 0.009), and the change in EI was smaller in group A than in the control group (41.09 vs. 66.42, *p* = 0.01). There were no significant differences in the changes in skin hydration or melanin index. Table 3 presents the adjusted change values based on covariate correction, along with the corresponding statistical results.

**TABLE 3:** Comparison results of skin parameters changes from baseline using ANCOVA or Quade nonparametric ANCOVA.

Skin parameters	Mean changes from baseline		Difference between groups	SE	*p*
	A	B			
Erythema index	—	—	—	—	0.01*
Melanin index	‐4.974^a1^	0.563^a1^	−5.537	5.982	0.358
TEWL	−0.734^a2^	2.195^a2^	−2.929	1.086	0.009**
Skin hydration	−4.373^a3^	−6.157^a3^	1.784	3.116	0.569

*Note:* *p*‐values were derived from ANCOVA or Quade Nonparametric ANCOVA (depending on the distribution of the effect size) and were adjusted for multiple comparisons: Bonferroni. a1: Covariates appearing in the model are evaluated at the following values: MI_0 = 96.0222; a2: Covariates appearing in the model are evaluated at the following values: TEWL_0 = 17.5080; a3: Covariates appearing in the model are evaluated at the following values: HYD_0 = 55.7807. “—”: Missing value indicate that this comparison uses Quade Nonparametric ANCOVA and there is no exact estimated marginal value.

Abbreviations: SE, standard error; TEWL, transepidermal water loss.

* *p* < 0.05.

** *p* < 0.01.

### 3.4 Subjective Assessment Endpoint Comparison

**FIGURE 5:**
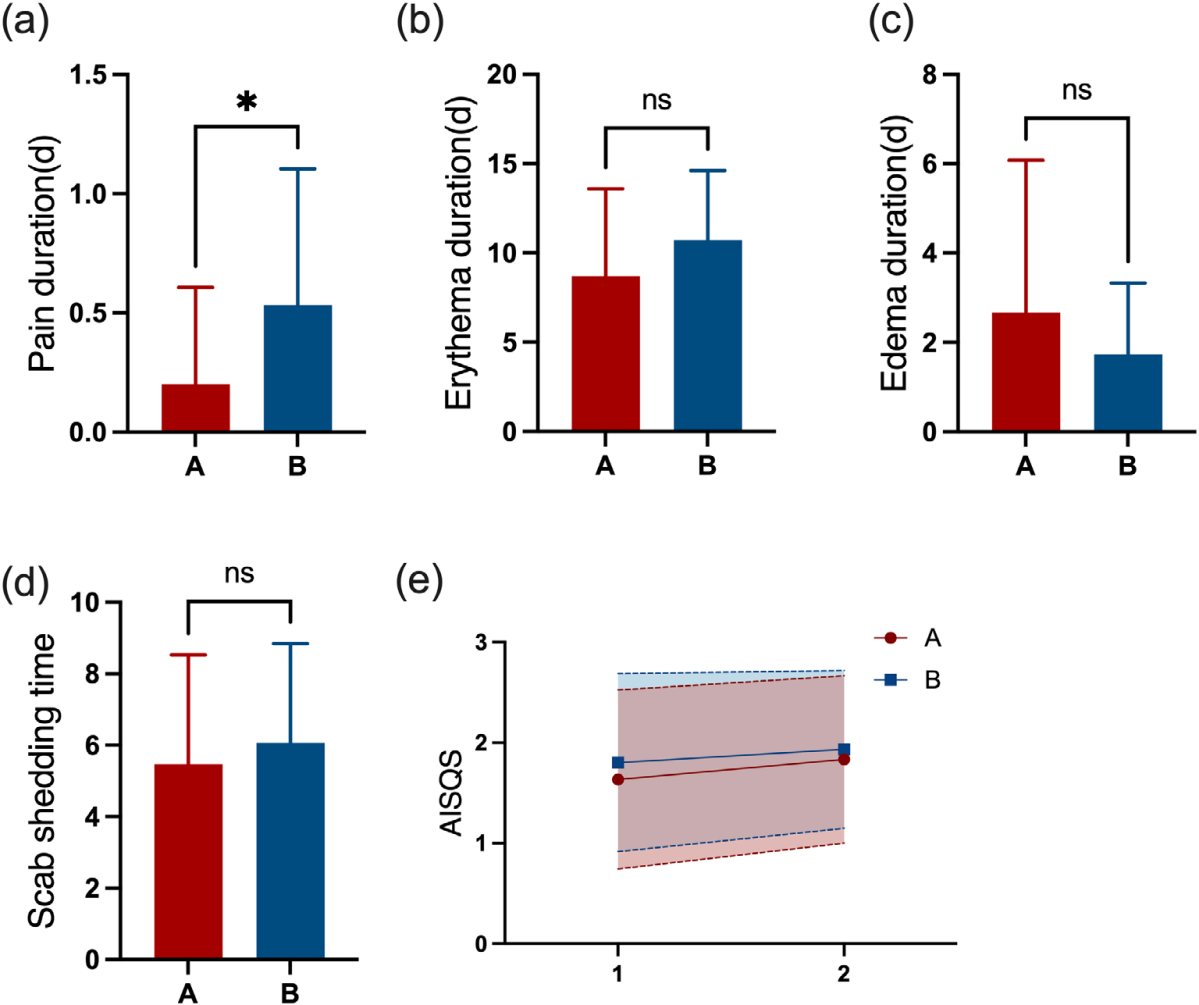
Objective evaluation results during follow‐up. (a) Pain duration; (b) Erythema duration; (c) Edema duration; (d) Scab shedding time; (e) AISQS, semi‐quantitative score of acute inflammatory response, which was assessed by clinician.

Group A showed a significantly shorter duration of pain (days) (0.2 vs. 0.53, *p* = 0.022) postoperatively, suggesting that hyaluronic acid may effectively alleviate postoperative pain. Although Group A had lower mean values for erythema duration (days) (8.70 vs. 10.70, *p* = 0.09) and scab shedding time (days) (5.48 vs. 6.07, *p* = 0.430) compared to Group B, these differences did not reach statistical significance. There were also no significant differences in edema duration, acute inflammatory semi‐quantitative scores of acute inflammation at immediate and 2‐h postoperative time points (Figure 5).

### 3.5 Patient Satisfaction and Safety Evaluation

**FIGURE 6:**
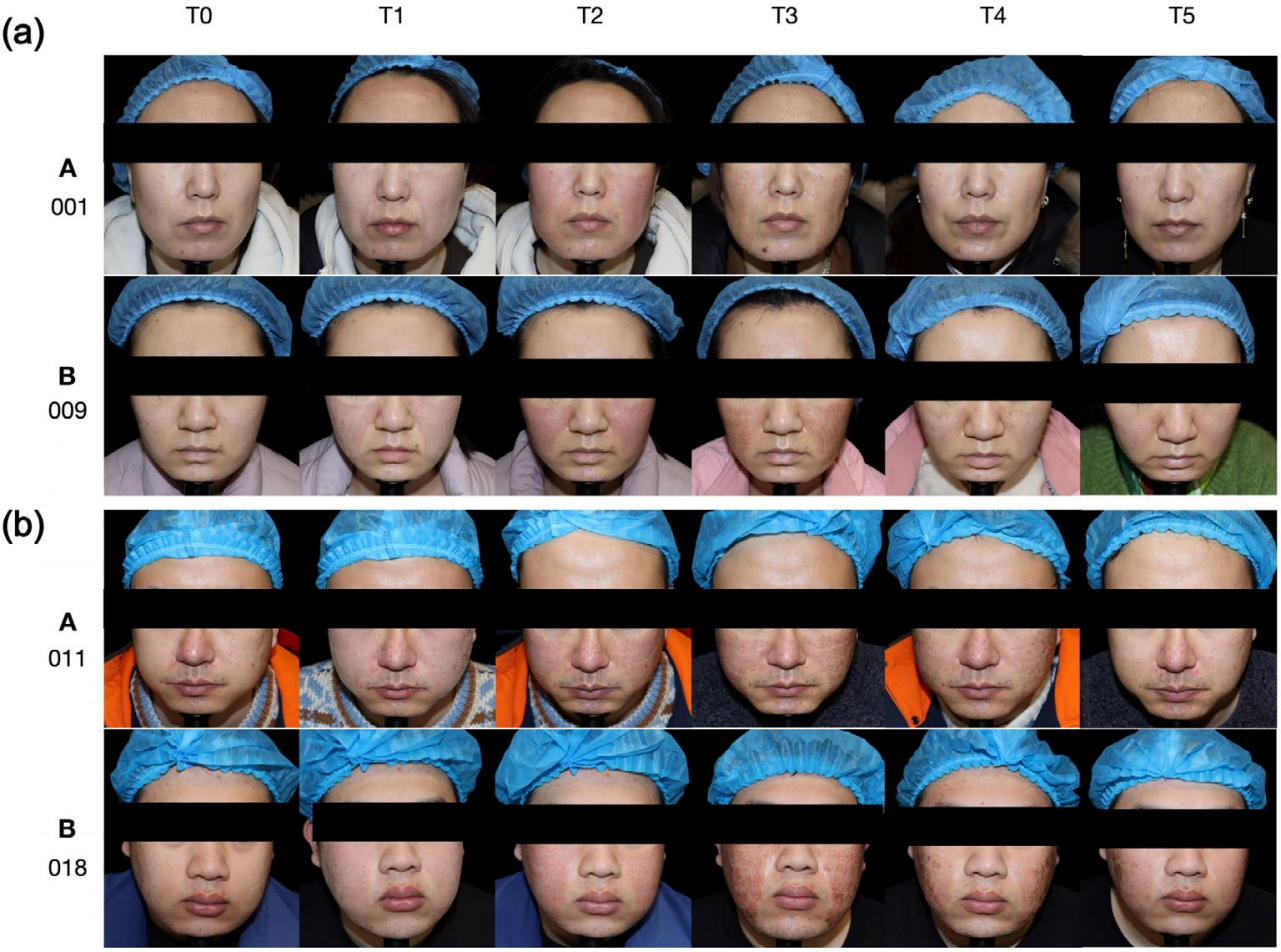
Comparison of clinical photos of the Group A (HA) and Group B (placebo). (a) Female. (b) Male. T0: Baseline; T1: Day 0 (immediate postoperative); T2: Day 0 (2 h after the skin care regimen); T3: Day 3; T4: Day 7; T5: Day 14.

**FIGURE 7:**
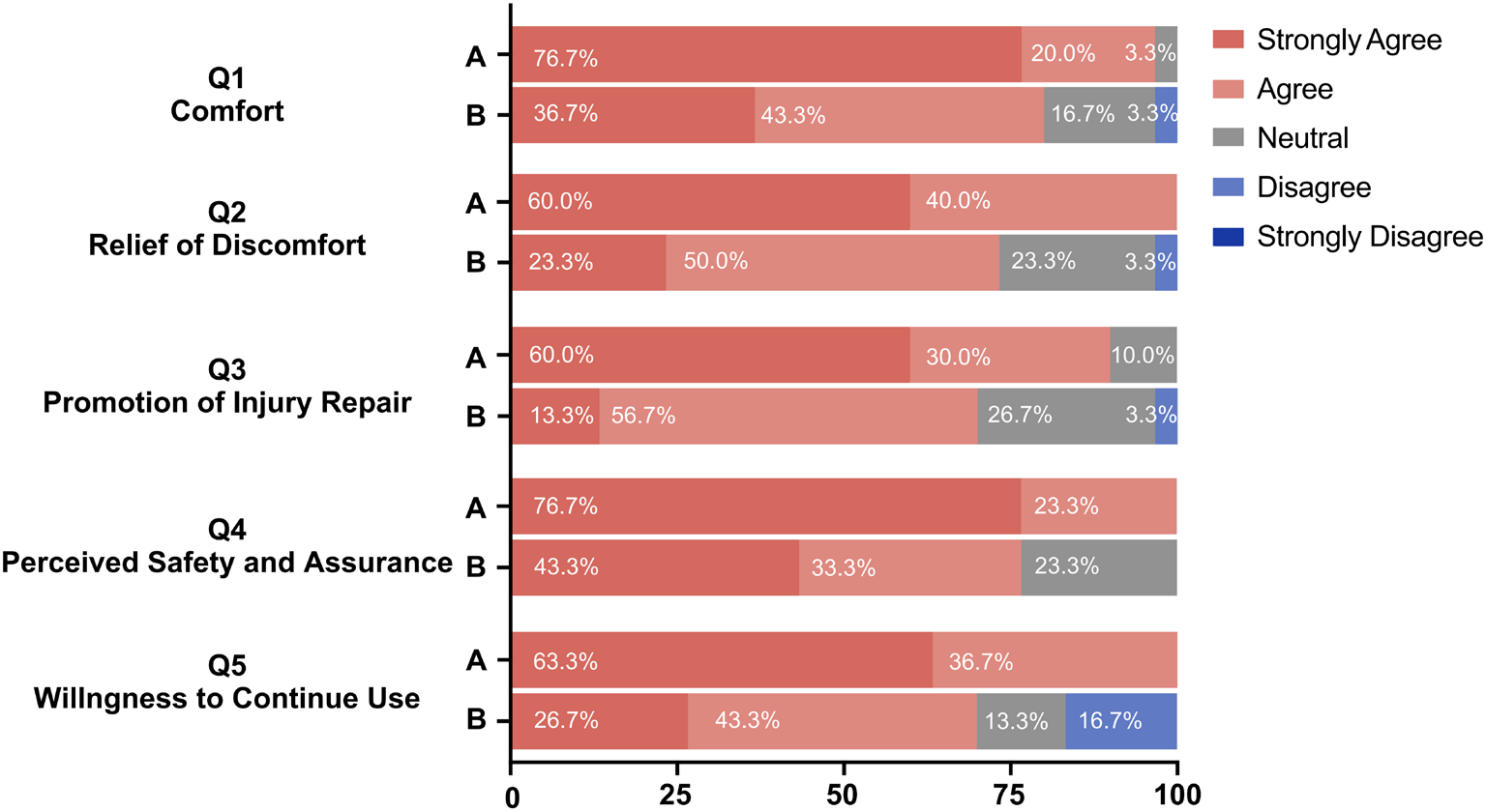
Patients' satisfaction 14 days after ablative fractional CO_2_ laser treatment. Question 1: I find this dressing makes me feel comfortable when using it; Question 2: I think this dressing can quickly and effectively relieve the discomfort after laser treatment; Question 3: I think this dressing can effectively promote damage repair after laser treatment; Question 4: I think this dressing makes me feel safer and more at ease; Question 5: I would like to use this dressing for my daily skincare routine or other post‐facial treatment.

Figure 6 presents a comparison of clinical photographs between Group A and Group B throughout the study. Based on the questionnaire feedback completed by participants on day 14, Group A demonstrated higher overall satisfaction, with better results in terms of comfort, relief of discomfort, skin repair promotion, feeling of safety during use, and willingness to use the product again compared to Group B (Figure 7).

No adverse events (AEs) or serious adverse events (SAEs) were reported in this study.

## 4 Discussion

This study represents the first prospective randomized controlled trial in China to evaluate the postoperative recovery efficacy and safety of composite hyaluronic acid dressings containing multiple molecular weights for CO_2_ fractional laser treatment. The results show that the composite hyaluronic acid formula has significant efficacy in improving postoperative transepidermal water loss (TEWL), erythema index, and pain duration.

Hyaluronic acid (HA), as an important natural moisturizing factor, has been widely used in dermatology, particularly for wound healing, anti‐aging, and skin barrier restoration. Bourguignon et al. confirmed that HA can regulate keratinocyte differentiation and the formation of extracellular lipid layers via interaction with CD44, maintaining epidermal barrier function [21]. The moisturizing effects of HA vary by molecular weight: HMW‐HA forms a protective film on the skin's surface, trapping moisture and preventing water loss, while LMW‐HA penetrates the epidermis for deeper hydration [22]. Compared to other moisturizers (e.g., polyethylene glycol, glycerin, ethylene glycol, and sorbitol), the advantage of HA is that it is not affected by relative humidity and has excellent water retention properties under both high and low humidity conditions [23].

Our experiment revealed a significant difference in TEWL between the two groups after 14 days. Additionally, the skin hydration levels also differed between the groups, with the HA Group showing an increase compared to the placebo group, although this difference was not statistically significant. While skin hydration and TEWL reflect different aspects of skin physiology, skin hydration indirectly assesses the stratum corneum (SC) structure and barrier function by measuring water content. TEWL, on the other hand, more closely reflects functional skin characteristics related to SC structural integrity, diffusion rates, and inflammatory conditions [24]. A reciprocal relationship between skin hydration and TEWL has been observed in other studies [24, 25].

The use of a composite molecular weight HA dressing significantly improved facial erythema following CO_2_ fractional laser treatment, which may be closely related to the regulation of skin healing, angiogenesis, and inflammatory responses by hyaluronic acid at different molecular weights. Erythema is a common post‐procedural reaction after laser treatment. HA plays a crucial role in regulating angiogenesis, acting as an effective modulator of endothelial cell (EC) function. HMW‐HA exhibits anti‐angiogenic effects, inhibiting EC proliferation, migration, and capillary formation [26]. HMW‐HA also enhances Foxp3 expression in Tregs, promoting anti‐inflammatory cytokines such as IL‐2, IL‐10, and TGF‐β. In contrast, LMW‐HA promotes angiogenesis by binding to Toll‐like receptors (TLR2 and TLR4), stimulating the production of TNF‐α and interleukins, displaying pro‐inflammatory characteristics [17, 27, 28, 29]. A clinical trial demonstrated the efficacy of a 0.2% sodium hyaluronate cream in treating rosacea, improving erythema, with better tolerance and compliance [30]. Our results showed significant improvement in the erythema index at all three follow‐up points in the HA group, suggesting HA's role in regulating angiogenesis, inflammation, and antioxidant activity, though further validation is needed.

In addition to its moisturizing and anti‐inflammatory properties, HA demonstrates notable analgesic effects. HA modulates the transient receptor potential vanilloid 1 (TRPV1) channel, reducing nociceptor activity and pain signaling [31]. Gomis et al. demonstrated that HA with a molecular weight above 40 kDa provides analgesia, with 860 and 2300 kDa HA offering sustained and efficient pain relief through HA receptor interactions [32]. Our results also showed that HA significantly reduced pain duration following CO_2_ fractional laser treatment.

Overall, the synergistic effects of HA with different molecular weights contribute to moisturizing, anti‐inflammatory, angiogenesis modulation, healing acceleration, and analgesia. HMW‐HA reduces inflammation, stabilizes the extracellular matrix, and inhibits unnecessary angiogenesis, while LMW‐HA improves local blood flow by promoting angiogenesis and accelerating the healing process.

Although there are many postoperative repair products available, some still have limitations when compared to hyaluronic acid (HA). For example, prolonged use of corticosteroids may lead to acneiform eruptions, and they are only recommended for use within 2 days post‐surgery [33]. Mineral water, while providing some soothing effect, has relatively weak moisturizing capabilities. Certain peptide‐based products, with larger molecular weights, may not be as easily absorbed as HA. Recombinant human epidermal growth factor (rhEGF) has strict storage requirements and may trigger allergic reactions. Therefore, HA has attracted significant attention due to its high hydrophilicity, good biocompatibility, and ease of use. Preliminary pilot studies have shown that antimicrobial peptides (AMPs) combined with HA‐based masks can effectively promote wound healing and reduce transient adverse reactions following fractional laser [34].

There are certain limitations in this study. First, the study's follow‐up duration was limited. Since changes in pigmentation often require a longer period to manifest, Takiwaki et al. also indicate that there is a positive correlation between erythema and melanin index, meaning that the more severe the erythema, the deeper the post‐inflammatory hyperpigmentation (PIH) [35], extending the follow‐up period may help further assess the impact of hyaluronic acid on pigmentation. Second, while the control group received a basic moisturizing cream to represent routine post‐procedural care, it should be noted that wet applications such as facial masks may themselves have therapeutic benefits in post‐laser skin recovery. As such, the lack of a wet dressing control could introduce potential confounding effects. Future studies will aim to refine the control interventions to enhance the rigor and generalizability of our findings. Third, patient self‐assessment of subjective outcomes may have introduced evaluation bias. There may be significant variations in pain perception, skin recovery, and subjective experiences among different patients, potentially affecting the accuracy of the results. Additionally, this study was not designed as a split‐face study. Although we used covariance analysis to adjust for potential confounders, individual differences cannot be entirely ruled out as a source of interference with the results.

Overall, this study suggests that composite hyaluronic acid with multiple molecular weights has a positive effect on skin recovery following fractional laser treatment, particularly in improving skin barrier function, reducing erythema, and alleviating pain. However, the effects of hyaluronic acid on hydration, pigmentation, and subjective recovery assessment were not significant. Future studies could explore the long‐term effects of hyaluronic acid treatment and assess its potential for use in combination with other dermatological therapies.

## 5 Conclusion

In conclusion, this prospective randomized controlled trial demonstrates that a skincare regimen with composite hyaluronic acid dressing containing multiple molecular weights improves skin barrier function, reduces erythema, and alleviates postoperative discomfort following CO_2_ fractional laser treatment, suggesting that its combined use with laser treatment is beneficial for promoting postoperative skin recovery.

## Author Contributions

Zongzhou Wu: Conceptualization, methodology, data curation. Xinlin Liang: Data curation, statistical analysis, visualization, writing – original draft preparation. Qian Yu: Writing – review and editing, supervision. Wei Zhang: Project administration, supervision. Yuling Shi: Supervision, funding acquisition.

## Ethics Statement

Reviewed and approved by the Ethics Committee of Shanghai Skin Disease Hospital (No. 2023–1 106 140 (scientific)). The patients in this manuscript have given written informed consent to the publication of their case details.

## Conflicts of Interest

The authors declare no conflicts of interest.

## Data Availability

The data that support the findings of this study are available from the corresponding author upon reasonable request.
